# Domesticated *gag* Gene of *Drosophila* LTR Retrotransposons Is Involved in Response to Oxidative Stress

**DOI:** 10.3390/genes11040396

**Published:** 2020-04-06

**Authors:** Pavel Makhnovskii, Yevheniia Balakireva, Lidia Nefedova, Anton Lavrenov, Ilya Kuzmin, Alexander Kim

**Affiliations:** 1 Laboratory of Exercise Physiology, Institute of Biomedical Problems of the Russian Academy of Sciences, Moscow 123007, Russia; maxpauel@gmail.com; 2 Department of Genetics, Faculty of Biology, Lomonosov Moscow State University, Moscow 119234, Russia; balakireva.evgesha@mail.ru (Y.B.); lidia_nefedova@mail.ru (L.N.); overtaki@mail.ru (A.L.); kuzmin.ilya@gmail.com (I.K.)

**Keywords:** *Drosophila*, LTR retrotransposon, molecular domestication, evolution, stress

## Abstract

*Drosophila melanogaster* is one of the most extensively used genetic model organisms for studying LTR retrotransposons that are represented by various groups in its genome. However, the phenomenon of molecular domestication of LTR retrotransposons has been insufficiently studied in *Drosophila*, as well as in other invertebrates. The present work is devoted to studying the role of the domesticated *gag* gene, *Gagr*, in the *Drosophila* genome. The *Gagr* gene has been shown to be involved in the response to stress caused by exposure to ammonium persulfate, but not in the stress response to oligomycin A, zeomycin, and cadmium chloride. Ammonium persulfate tissue specifically activates the expression of *Gagr* in the tissues of the carcass, but not in the gut. We found that the *Gagr* gene promoter contains one binding motif for the transcription factor kayak, a component of the JNK signaling pathway, and two binding motifs for the transcription factor Stat92E, a component of the Jak-STAT signaling pathway. Remarkably, *Gagr* orthologs contain the second binding motif for Stat92E only in *D. melanogaster*, *D. simulans* and *D. sechellia*, whereas in *D. yakuba* and *D. erecta*, *Gagr* orthologs contain a single motif, and there are no binding sites for Stat92E in the promoters of *Gagr* orthologs in *D. ananassae* and in species outside the melanogaster group. The data obtained indicate the formation of the protective function of the *Gagr* gene during evolution.

## 1. Introduction

Despite their selfish nature, retroelements are a powerful genetic resource for the host organism and can play a significant evolutionary and adaptive role in eukaryotes [1,2,3]. There are various mechanisms by which new genes arise; one of them is molecular domestication of retroelement (retrotransposon or retrovirus) sequences [4]. The term “molecular domestication” was first proposed by Wolfgang Miller in 1997 to describe the phenomenon of the adaptation of the sequences of mobile elements by the organism for its own benefit [5]. The most important concept applicable to the evolutionary role of sequences of mobile elements is “exaptation”. This term implies the formation of new genetic structures and functions that, as a rule, appeared as a result of the long evolution of sequences of domesticated mobile elements [4,6].

The most studied cases of molecular domestication/exaptation of retroelements are the domestication of retrotransposons with long terminal repeats (LTR retrotransposons) and endogenous retroviruses. For all three genes of LTR retroelements, *gag*, *pol*, and *env*, domesticated homologs with functions beneficial for the host organism have been discovered. The greatest variety of such genes has been found for homologs of the capsid gene, *gag* [3]. Examples of *gag* domestication in mammals are represented by the gene families MART (Mammalian RetroTransposons), or SIRH (Sushi-Ichi Retrotransposon Homologues), and PNMA (Paraneoplastic Ma antigens) [4]. The MART/SIRH and PNMA families originate from independent domestications of LTR retroelements (in the first case, from the sushi-ichi group, in the second, from the *Ty3/Gypsy* group) [7,8]. Many genes of the MART family are expressed in the placenta, and some are necessary in the early stages of placenta formation and its development [9,10]. On the other hand, among the genes of the PNMA family there are apoptosis regulators [11]. Another example of successful *gag* domestication is the emergence of the SCAN domain which is widely represented among the transcription factors of Tetrapoda. SCAN originated as a result of the domestication of an LTR retrotransposon of the Gmr1-like group about 300 Ma ago [12,13]. Many of the genes that have the sequence encoding the SCAN domain of transcription factors that control various biological processes: embryonic development, hematopoiesis, metabolism, etc. [4]. Of particular interest is the *gag* homolog with a specific antiviral function, *Fv1* (friend-virus-susceptibility-1), which is a factor limiting exogenous mouse leukemia virus (MLV) and other types of retroviruses. *Fv1* is derived from the *gag* gene of the endogenous retrovirus MERV-L [14,15,16].

The molecular domestication of LTR retrotransposons in invertebrates is poorly understood. In *Drosophila melanogaster*, the host *Gagr* gene (Gag-related protein) is described as a homologue of the *gag* gene of LTR retrotransposons of the Gypsy group. At present, the specific function of *Gagr* is unknown and little is known about the regulation of its expression. Orthologs of this gene are found in all sequenced genomes of the *Drosophila* genus; they possess a highly conservative structure and are the result of long-term domestication [17]. There are several research results for the *Gagr* gene that indirectly indicate its involvement in a number of important processes related to stress reactions. *Gagr* expression is activated in response to the induction by bacterial lipopolysaccharides in S2 cells, and this activation depends on the regulators of the MAPK/JNK stress signaling pathways *Tak1*, *hep* and *bsk* [18]. *Gagr* expression increases significantly after intraabdominal injection of DCV viruses (*Drosophila* C virus), FHV (Flock House virus) and SINV (Sindbis virus) [19]. To predict the function of the Gagr protein, its interactions with other proteins that were established during the identification of protein–protein complexes in S2R+ *D. melanogaster* cells are of importance [20]. At least three Gagr partners, 14-3-3epsilon, Pdi and eIF3j, are involved in stress-related functions. 14-3-3epsilon is a conservative regulator of the activity of MAPK and other stress signaling pathways in animals [21]. Chaperone Pdi plays an important role in endoplasmic reticulum stress (ER-stress) and UPR (unfolded protein response) [22,23]. eIF3j is a subunit of translation initiation factor. eIF3j has been shown to be necessary for IRES-dependent translation which occurs under conditions of cell stress [24]. Thus, it is necessary to study the role of the *Gagr* gene in cell stress, taking into account the known data on its activation and protein–protein interactions. This will reveal new functions and evolutionary capabilities that are provided by domestication of LTR retroelements in eukaryotes.

## 2. Materials and Methods

### 2.1. Drosophila Strains and Treatments

*D. melanogaster* Canton-S strain was used in all experiments. Fly stocks were maintained in a standard nutrient agar medium at 25 °C. Adult flies 6–8 days old were used in all experiments.

Four stress agents were used in the study: ammonium persulfate, oligomycin A, cadmium chloride or zeocin. To induce stress, flies were placed in test tubes with a nutrient medium containing the active substance, and after 24 h, flies or organs were collected to isolate total RNA. As controls, intact flies incubated with an equivalent amount of solvent or appropriate salt in the feed without an active agent were used, depending on the effect (Table 1). In the experiments, cadmium chloride (SigmaAldrich, St. Louis, MO, USA, cat#202908) was used as an inducer of endoplasmatic reticulum (ER) stress; oligomycin A (SigmaAldrich, USA, cat#75351) was used as an inducer of mitochondrial stress; zeocin (Thermo Fisher Scientific, Waltham, MA, USA, cat#R25001) was used as an inducer of stress associated with DNA damage; ammonium persulfate (Thermo Fisher Scientific, USA, cat#17874) was used as an inducer of oxidative stress (Table 1). The concentrations of the stress agents were selected based on the observed mortality (~ 5-10%) after 24 h of exposure. The start time of the experiment was chosen, taking into account the possible influence of circadian factors, nutrition status and light mode. The degree of stress was evaluated by *upd3* cytokine expression level.

In experiments to study the temporal dynamics of gene expression in response to ammonium persulfate, exposure periods of 6, 12, 24, 30 h were used. After 30 h exposure, the flies were transferred to a favorable environment and kept for 2 and 12 h. In the experiments, a 24 h exposure to 0.1 M ammonium persulfate and an intact control were used to evaluate gene expression in organs.

### 2.2. RNA Isolation and Quantitative RT-PCR

RNA was isolated from pools (five females, seven males or 20 separate organs) in 5–7 biological replicates using the ExtractRNA reagent (Evrogen, Moscow, Russia), according to the manufacturer’s protocol, then it was treated with DNase I (Thermo Fisher Scientific, USA). Reverse transcription was carried out using an MMLV-RT kit (Evrogen, Russia), according to the manufacturer’s protocol, with random primers (Evrogen, Russia).

For quantitative PCR with the obtained cDNA, a Taq polymerase-based reaction mixture with intercalating dye SYBRGreen I (Evrogen, Russia) was used in accordance with the manufacturer’s protocol. The reaction was performed using a Mini Opticon Real-Time PCR System (Bio-Rad Laboratories, Hercules, CA, USA). The relative expression of the genes *Gagr*, *vir*-*1*, *Relish*, *upd3*, *hid*, *sid*, *Tspo*, and *Hsp22*, normalized to the expression of three reference genes, *αTub84D*, *RpL40*, *EloB* («2^ΔCt^» method), was analyzed. Amplification was performed with primers shown in Table 2.

### 2.3. Microarray Data Reprocessing and Co-Expression Network Analysis

**Figure 1:**
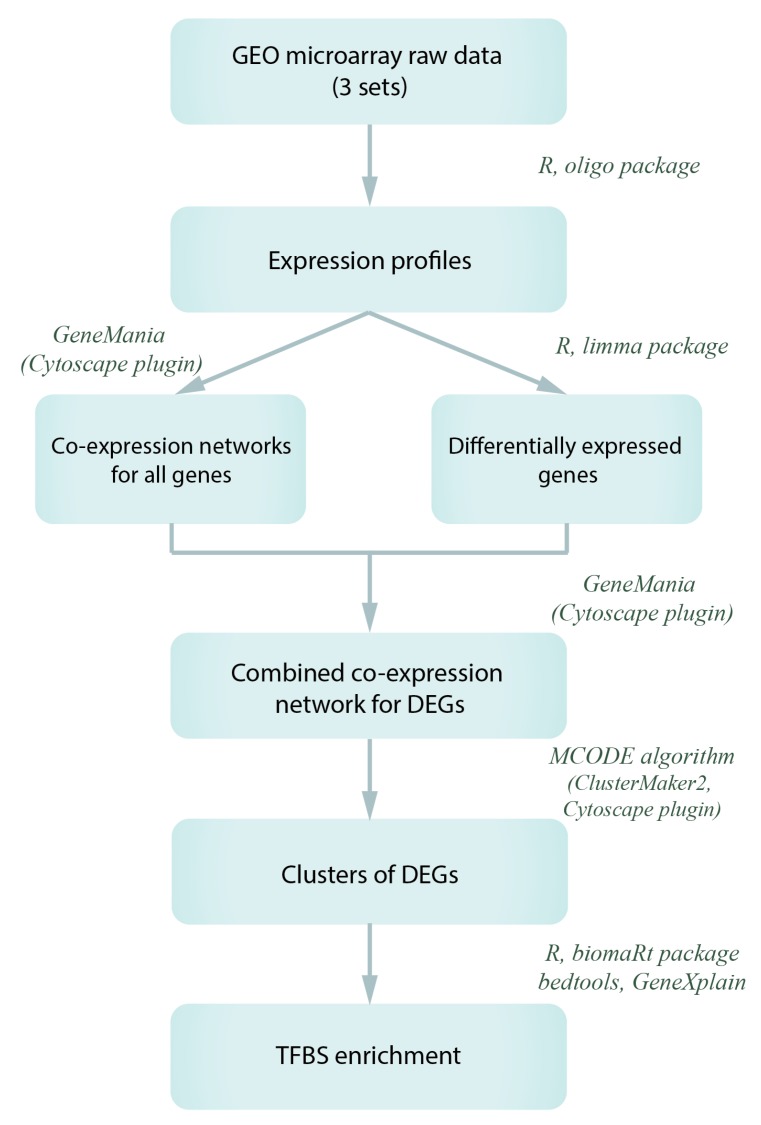
Workflow for the search and analysis of co-expression groups for the *Gagr* gene and genes with virus-induced expression. DEGs—differently expressed genes; TFBS—transcription factor binding sites.

To search for co-expressed genes, we used three sets of transcriptomic data (DNA microarrays), in which the activation of *Gagr* gene expression is observed. The analysis used the data presented in the GEO database: GSE2828 (all samples), GSE31542 (all samples), GSE42255 (all samples, except sugar treatment and young flies). All raw data were reprocessed by oligo and limma R packages (rma normalization, empirical Bayes statistics for differential expression, separate multiple testing model with a cutoff score for P_adj_ (Benjamini-Hochberg correction) < 0.05 and for Fold Change > 2). For each of three expression profiles we constructed a co-expression network by GeneMania algorithm (v.3.5.1, Cytoscape plugin). Furthermore, these networks were used to construct a network for genes demonstrating differential expression under the same conditions (at least in one) as the *Gagr* gene. Using the MCODE algorithm (ClusterMaker2 plugin of Cytoscape, options—‘Haircut’, ‘Fluff’, Node Score Cutoff = 0.2), we obtained eight co-expression clusters. The bioinformatic analysis scheme is shown in Figure 1.

### 2.4. TFBS Enrichment Analysis and Search for Potential Transcription Regulators of Gagr and Co-Expressed Genes 

To find potential regulators for each co-expression cluster, we performed TFBS enrichment in the promoter regions of selected genes relative to a random sample of 5000 protein-coding genes. Using the biomaRt package of the R programming software (https://bioconductor.org/packages/release/bioc/html/biomaRt.html) and the bedtools (https://bedtools.readthedocs.io/), the promoter regions (from –1000 bp to +100 bp relative to transcription starts) were isolated for all genes. The search for binding sites was performed with the positional weight matrices of the Transfac 2019.2 database (insects) using the GeneXplain platform (function “Search for enriched TFBSs (tracks)”) [34]. To search for significantly enriched weight matrices for each group of promoters, the Fisher test (random value is the number of promoters in which at least one binding site for a specific transcription factor is present) and the binomial test (random value is the site frequency per 1000 bp of a promoter) were performed. The maximum adjusted fold enrichment (statistically corrected odds ratios with a 99% confidence interval) was determined for each matrix (with maximum site frequency of one per 2000 bp). Adjusted fold enrichment >1 and FDR <0.05 for the binomial test were set as denoting significantly enriched TFBSs. The search for TFBSs in the *Gagr* gene promoter was carried out in the region of -1000 bp to + 100 bp (the threshold PWM score was set to the minimum site frequency of 1 per 2000 bp).

### 2.5. Alignment of The Promoter Regions of The Gagr Gene of Various Drosophila Species and Phylogenetic Analysis

Alignment of nucleotide sequences was carried out using the Muscle tool (https://www.ebi.ac.uk/Tools/msa/muscle/). The promoter regions of *Gagr* orthologs limited to conservative regions in the studied species were identified. These regions correspond to the coordinates of –881 bp to +119 bp in the TSS *Gagr-A* transcript for *D. melanogaster*. The phylogenetic trees were built by the maximum likelihood method using the UGENE program (http://ugene.net/): substitution model—HKY85, bootstrap—1000 replicates.

## 3. Results

### 3.1. The Influence of Exogenous Stress Factors Causing Cell Homeostasis Disturbance on The Expression of The *Gagr* Gene in *D. melanogaster* Imago

Activation of *Gagr* transcription is associated with both non-specific viral exposure [19] and the influence of other biotic stress factors, such as bacterial injection of *Bacillus cereus* [35] or bacterial lipopolysaccharides [18]. Such regulation of expression may be associated with specific immune response, or with the activation of universal stress signaling cascades (JNK, MAPK or Jak-STAT) due to impaired tissue/cell homeostasis. Therefore, we investigated the effect of various agents that damage biological macromolecules (proteins, DNA) and disrupt cell homeostasis (ammonium persulfate, oligomycin A, cadmium chloride and zeocin) on the expression of the *Gagr* gene in *D. melanogaster* Canton-S females. To assess the development of stress, we analyzed the expression of a number of known stress-induced genes in response to stress agents (Table 3).

**Figure 2:**
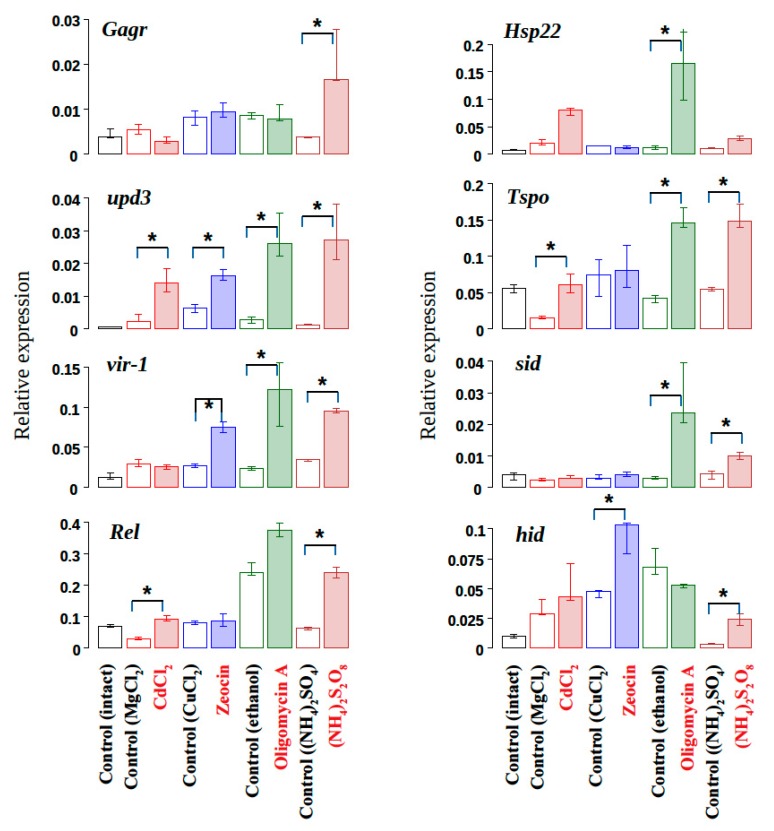
Analysis of the expression of *Gagr* and stress-induced genes in *D. melanogaster* females in response to various stress agents after 24 h exposure. *—significant differences in exposure relative to control (Mann-Whitney test, *p*-value < 0.01, N = 5-7). The data are presented as median and interquartile range.

The experiments showed that all the stressors lead to a significant activation of the expression of the stress-induced cytokine *upd3* compared to the control conditions (Figure 2). For other genes, specific activation by certain stresses was observed. The activation of *Gagr* gene expression (~4-fold) was observed only after a 24 h exposure to ammonium persulfate. It is noteworthy that exposure to ammonium persulfate also led to a significant increase in the expression of all used stress-induced genes compared to the controls. Exposure to zeocin, oligomycin A and cadmium chloride did not lead to changes in the level of *Gagr* expression compared to the control. 

The experimental results showed that *Gagr* expression can increase significantly not only in response to a viral infection [19], but also in response to abiotic stimuli leading to stress. We found that it depends on the specific stress caused by exposure to peroxo compounds (ammonium persulfate). The mechanism of action of persulfate on the cell is poorly understood, but its oxidative potential and the ability to initiate radical reactions suggest its nonspecific action on the cell and the induction of various oxidative stress reactions. The result of this effect may be the oxidation of biological macromolecules and the disruption of protein folding in various cell compartments (ER, cytoplasm, mitochondria) or the damage of cells and the development of a systemic stress response. However, when we studied the effect of other specific stress agents that disrupt cell homeostasis, we did not detect the activation of *Gagr* expression. Mitochondrial stress caused by oligomycin A was accompanied by a significant activation of expression of the stress-induced genes studied, but did not lead to *Gagr* activation. This result suggests that the signaling mechanisms that are activated during mitochondrial stress are insufficient or do not play a decisive role in the stress-induced activation of *Gagr*. Little is known about the mechanisms of mitochondrial stress development and implicated signaling pathways in *Drosophila*. Nevertheless, for other model organisms, the JNK cascade and Atf4 transcription factor were shown to be involved in the response [48,49]. Given this fact, the activation of the JNK cascade can be assumed to be insufficient for the stress-induced activation of the *Gagr* gene, which is likely to occur via other specific mechanisms. To evaluate the effect of ER stress on *Gagr* activation, we used cadmium chloride. Despite a high concentration of cadmium chloride (0.1 M) in the nutrient medium, we observed a more specific and less significant activation of the expression of the studied stress markers in comparison with the effect of oligomycin A or ammonium persulfate, whereas *Gagr* expression was not affected by cadmium salts. Thus, the ER stress caused by such exposure is not crucial or sufficient to activate *Gagr* expression. The experiment with zeocin showed significant activation of the *hid* gene, a target of the p53 cascade activated by DNA damage. But activation of *Gagr* expression is probably independent of the stress associated with such DNA damage.

### 3.2. Investigation of The Temporal Dynamics of Expression of *Gagr* Gene and Stress Response Effector Genes Upon Exposure to Ammonium Persulfate in *D. melanogaster* Imago

**Figure 3:**
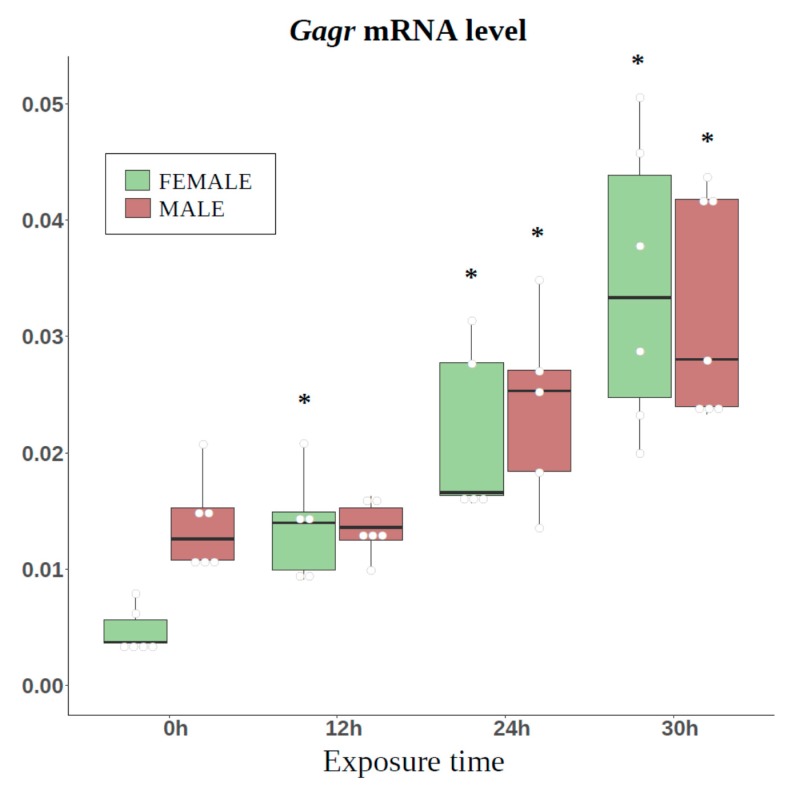
Activation of *Gagr* expression of *D. melanogaster* females and males in response to different times of exposure to 0.1 M ammonium persulfate. *—significant differences relative to the basal level (Mann-Whitney test, *p*-value < 0.01, N = 5–7). The data are presented as scatterplots and boxplots (median, interquartile range, 5 to 95 percentile).

We found that ammonium persulfate activates *Gagr* expression after 24 hours of exposure in females. It is known that the response to stressful stimuli (e.g., starvation or oxidative stress) may be sex-specific in *Drosophila* [50]; therefore, we evaluated how different times (12, 24, and 30 h) of exposure to 0.1 M ammonium persulfate affect *Gagr* expression in females and males. In females, the level of *Gagr* mRNA was shown to increase after 12 h, while in males at this time point it remained at the basal level, increasing after 24 and 30 h exposure. Moreover, the differences in the level of *Gagr* mRNA in females and males are observed only at the basal level (Figure 3). These data indicate different mechanisms of activation of *Gagr* expression during stress development. Besides, the female-specific activation of *Gagr* expression was observed after Kallithea virus infection [51].

**Figure 4:**
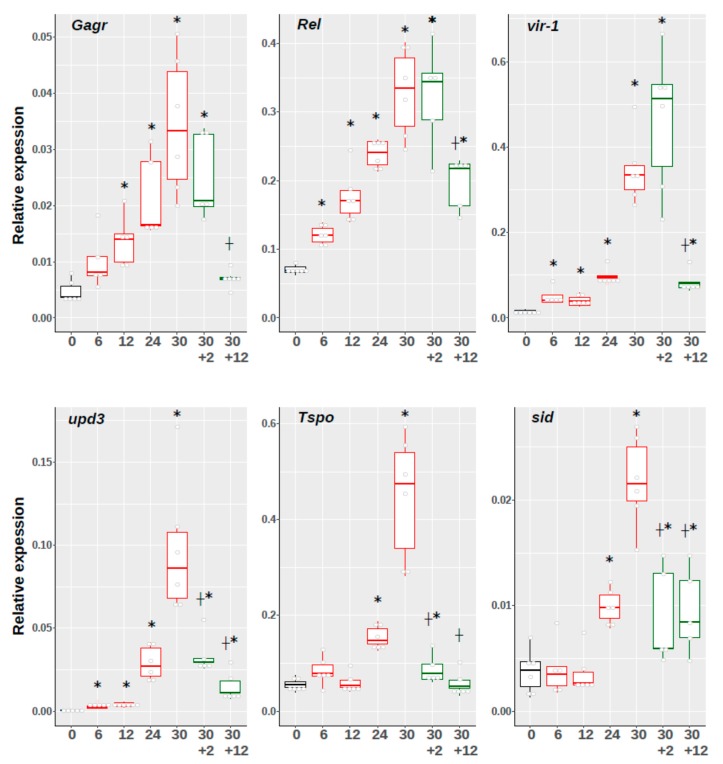
Relative expression of *Gagr* and stress-induced genes in *D. melanogaster* females in response to different times of exposure to 0.1 M ammonium persulfate and after 30 h exposure followed by 2 or 12 h recovery periods. The horizontal axis shows the exposure time, 2 and 12 h recovery periods are marked on the axis as “30 + 2” and “30 + 12”. *—significant differences relative to the basal level, **┼**—significant differences after the recovery period relative to the level after 30 h (Mann-Whitney test, *p*-value < 0.01, N = 5–6). The data are presented as scatterplots and boxplots (median, interquartile range, 5 to 95 percentile).

We observed the significant activation of *Gagr* expression after 30 hours of exposure to ammonium persulfate. However, it is not clear what this activation is associated with: direct accumulation of a stress agent or with long recovery processes that are not dependent on the presentation of a stress factor. To find out, we evaluated how different times (6, 12, 24, and 30 h) of exposure to ammonium persulfate to see if this would affect the expression of the *Gagr* gene and stress-induced genes in females. In addition, after 30 h exposure, the flies were transplanted into a favorable environment and gene expression was evaluated after a short (2 h) and long (12 h) recovery period. The mRNA levels of the *upd3*, *vir-1* and *Rel* genes were found to be significantly increased after a 6 h exposure relative to the basal levels, significantly increasing after 24 and 30 h. On the other hand, the mRNA levels of the *Tspo* and *sid* genes (proapoptotic) were significantly higher than the basal levels only after 24 and 30 h exposure. Moreover, a short recovery period (2 h) was shown to lead to a significant decrease in the expression of *Tspo* and sid, as well as the cytokine *upd3* genes, while the *Gagr*, *vir-1* and *Rel* mRNA levels remained high, but significantly decreased after a long recovery period relative to “30 h” (*Gagr* expression decreased to basal level) (Figure 4).

Thus, we identified the phase in which significant systemic stress develops (after 24 h exposure). This phase of stress development is characterized by a significant activation of the transcription of the effector genes *vir-1*, *upd3* and *Rel*, as well as the genes for proapoptotic factors: the *sid* gene (stress-induced DNase) and the *Tspo* gene (mitochondrial translocon). The *Gagr* gene is also significantly activated precisely in this phase: its expression increases almost tenfold after 30 h exposure to ammonium persulfate. We found that after a short recovery period (2 h) the *Gagr* mRNA is maintained at a high level. However, after a long recovery period (12 h), its expression becomes comparable to the basal level. These data indicate that *Gagr* function is necessary during stress development to a greater extent than during the recovery period; however, its mRNA level is not so dependent on the presence of the stress factor as the mRNA level of the proapoptotic genes *sid* and *Tspo*. Such diverse dynamics of expression in a short recovery period (2h) can be associated both with the specificity of the stress-related gene regulation and with the properties of different mRNAs (determinants of its stability or decay) under stress conditions.

### 3.3. Study of Tissue-Specific Activation of *Gagr* Gene Expression in *D. melanogaster* Females Induced by Ammonium Persulfate

**Figure 5:**
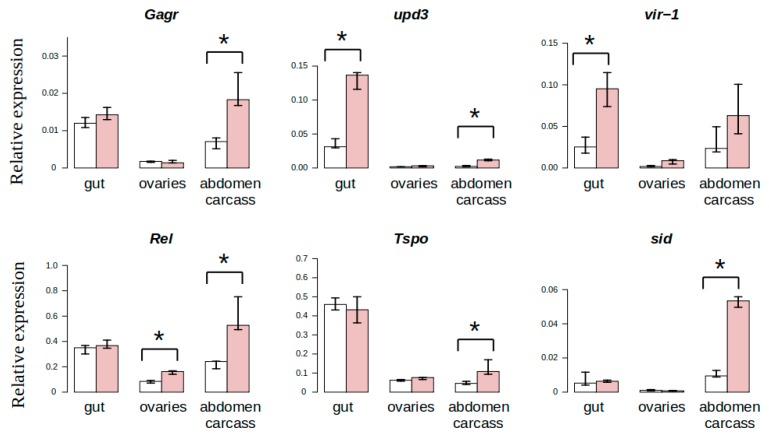
Tissue-specific activation of *Gagr* expression and stress-induced genes in *D. melanogaster* females in response to 24 h exposure to 0.1 M ammonium persulfate. White bars correspond to intact control; red bars correspond to the effects of ammonium persulfate. *—significant differences relative to control (Mann-Whitney test, *p*-value < 0.01, N = 5-6. The data are presented as median and interquartile range.

In order to evaluate in which tissues *Gagr* gene activation caused by peroxo compounds occurs, we experimentally examined the levels of *Gagr* mRNA and stress-induced genes in various tissues of females (ovaries, gut, and abdomen carcass) in response to 24 h exposure to 0.1 M ammonium persulfate. As a result of the experiment, ammonium persulfate was found to tissue-specifically activate the expression of *Gagr* and individual genes (*Rel*, *sid* and *Tspo*), their mRNA levels were significantly increased in the tissues of the carcass, but not in the gut relative to the intact control (Figure 5). Thus, *Gagr* expression in the gut and ovaries does not change, but in the tissues of the carcass increases. At the same time, the basal level of *Gagr* expression in the gut is high. Such tissue-specific expression is also characteristic for some of the stress-induced genes studied: *Tpso*, *sid*, and to a lesser degree for *Rel*. Observed tissue-specific regulation of the *Gagr* expression can be considered with the different status of stress-associated signaling pathways, as well as the functions of investigated tissues. The fat body, which makes up a significant part of the abdominal carcass, differs from gut in metabolic activity, specificity of humoral regulation and a set of expressed tissue-specific cytokines [52]. A systemic response develops in the fat body, in which a large number of mechanisms are involved, including upd3-dependent activation of the Jak-STAT signaling pathway [53]. Probably, some of these systemic or humoral mechanisms can provide stress-dependent activation of *Gagr* expression in the fat body. In turn, intestinal tissues may have the activated status of some stress-related pathways under intact conditions, which determines the absence of changes in Gagr expression in the gut. In particular, in this experiment, we observed a very high level of expression of *upd3* and *Tspo* in the gut relative to the carcass ones under intact conditions.

### 3.4. Search for Potential Regulators of Stress-Induced Activation of Expression of The *Gagr* Gene in *D. melanogaster* Imago

**Figure 6:**
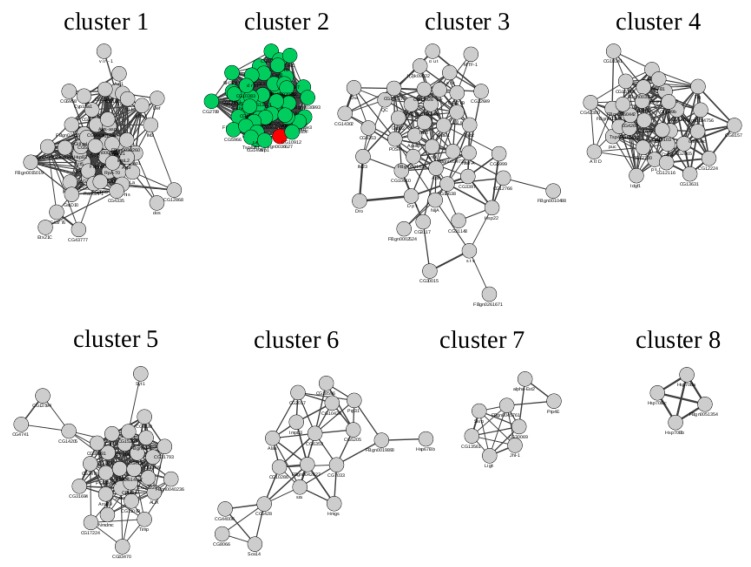
Co-expression clusters of genes with virus-induced expression (according to GEO: GSE2828, GSE31542, GSE42255). The genes in the cluster with *Gagr* (cluster 2) are marked in green, the *Gagr* gene is marked in red.

The study was based on the search for overrepresented TFBS in promoters of co-expressed with *Gagr* genes (relative to the reference set of promoters of randomly selected 5000 genes). In order to identify genes co-expressed with *Gagr* in *D. melanogaster* imago, we used transcriptional data on the effects of DCV (*Drosophila* C virus) [51], FHV (Flock house virus) and SINV (Sindbis virus) on gene expression in the adult male of the Oregon-R strain [19], as well as data on the influence of various stress factors (heat shock, ionizing radiation, hyperoxia, hydrogen peroxide, aging) on the transcriptome of adult males of the Oregon-R strain [54]. These microarray data were reprocessed, and expression profiles were used for co-expression network construction and for meta-analysis provided by GeneMania algorithm for selected genes. The final co-expression network was built for genes with differential expression under the same conditions as the *Gagr* gene. The co-expression network was divided into clusters by the graph-oriented MCODE algorithm (Figure 6; see network’s nodes and edges information in Table S1).

Overrepresented TFBS were found in the promoter regions for each gene cluster using the algorithms of the GeneXplain platform and the TRANSFAC database. For various clusters, TFBS enrichment for TF involved in stress and immune responses (ATF/CREB, AP1, GATA, STAT, Dif, Rel, Tfam, Hsf, and cnc) were found. The result of TFBS enrichment is presented in Table 4 (see full data in Table S2).

**Figure 7:**
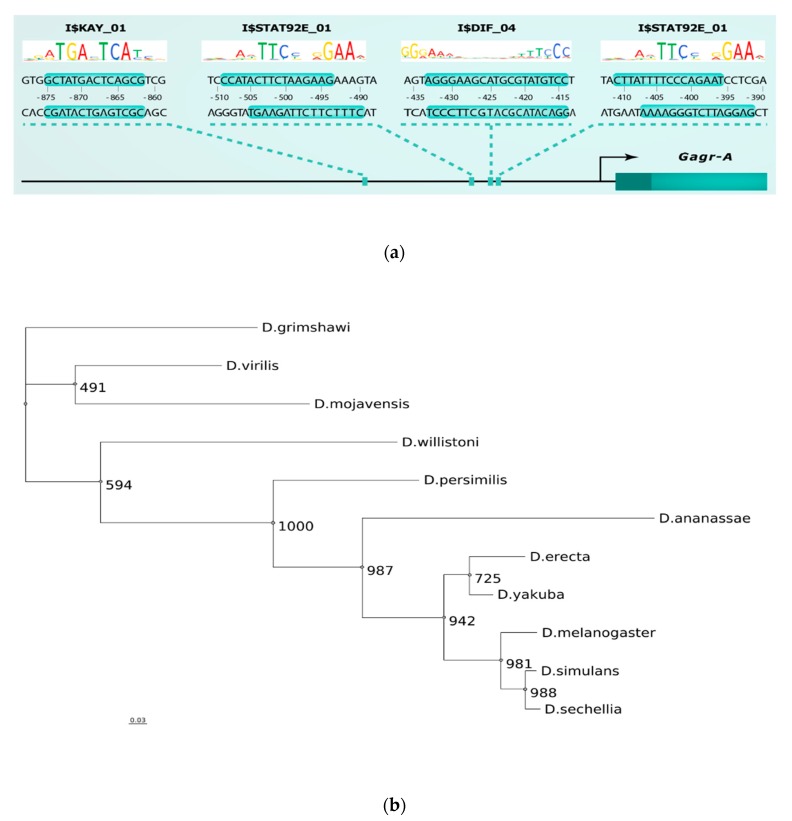
Analysis of the promoter regions of the *Gagr* gene and its orthologs. (**A**) Localization of binding sites for the transcription factors Dif, kay and Stat92E in the *Gagr* gene promoter. (**B**) Phylogram based on the promoter sequences of the *Drosophila Gagr* gene; bootstrap branch values are presented (the number of bootstrap replicas is 1000).

We found that different clusters were enriched for specific sets of TFBSs. For example, promoters of cluster 4 were enriched with motifs of the ER-stress-related TFs (Atf2, Atf6, CREBA) [55], and cluster 6 was characterized by enrichment with Heat shock factor and cnc motifs, which play an important role in unfolded protein response and oxidative stress [56] (Table 4). Additionally, we noted the activated status of the stress-related JNK cascade and transcription factors kay or Jra (enriched in clusters 1, 2, 4, 5; Table 4). The *Gagr* co-expression group (cluster 2) promoters were enriched with motifs of kayak, GATA family transcription factors, Stat92E, Dif, Rel, and Tfam (Table 4). We evaluated which of the motifs were present in the promoter of the *Gagr* gene (from −1000 to +100 bp relative to the start of transcription). We found one binding motif for kayak (JNK cascade), two binding motifs for Stat92E (Jak-STAT pathway), and one binding motif for Dif (Toll pathway) (Figure 7A). All these sites have a high PWM score. It is noteworthy that we identified these TFs as the most probable ones involved in the regulation of stress-induced genes co-expressed with the *Gagr* gene.

The JNK and Jak-STAT signaling pathways are the most important cascades in the early stages of regeneration after acute stress or injury [57]. The assumption of such regulation is in good agreement with the results of our experiments and other studies in which the activation of *Gagr* expression is observed in response to significant stress (viral infection, oxidative stress caused by peroxo compounds). It is also possible that the Toll signaling pathway may participate in the stress response in *D. melanogaster.*

### 3.5. Phylogenetic Analysis of Promoter Regions of The *Gagr* Gene in Various Species of The Genus Drosophila

To assess the functional and evolutionary significance of the detected motifs, we examined how conservative their presence and localization in the *Gagr* gene and its orthologs’ promoters. For study, we isolated the promoter regions of the *Gagr* gene orthologs in sequenced genomes of *Drosophila* species of the melanogaster group (*D. simulans*, *D. sechellia*, *D. melanogaster*, *D. yakuba*, *D. erecta*, *D. ananassae*) and other groups (*D. persimilis*, *D. willistoni*, *D. virilis*, *D. mojavensis*, *D. grimshawi*).

**Figure 8:**
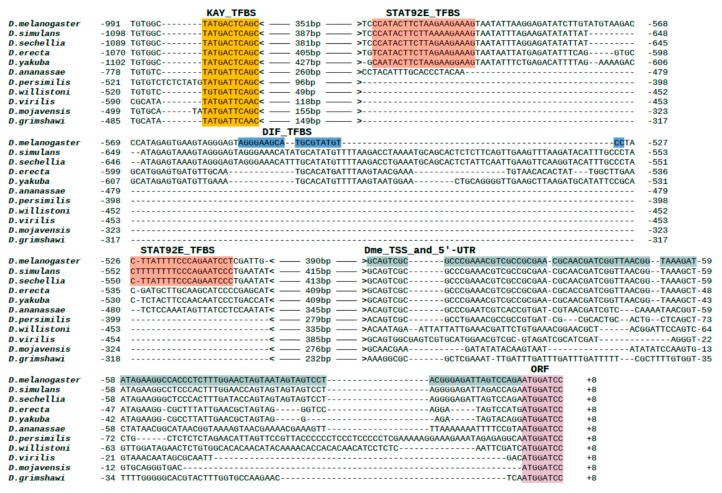
Alignment of *Gagr* promoter regions for different *Drosophila* species. Features marked in color: TFBS –Transcription factor binding sites; Dme_TSS_and_5’-UTR—Transcription start site and 5’-untraslated region of *D. melanogaster Gagr*; ORF—Open reading frame.

As a result of the analysis of the promoter regions, one motif I$KAY_01 was found in all species, whereas the motif I$STAT92E_01 is present only in species of the melanogaster group; the binding motif for Dif (I$DIF_04) is non-conservative and is found only in *D. melanogaster* (Figure 8; see full multiple alignment in File S1). We examined the localization of these motifs in a consensus alignment sequence and found that the motif I$KAY_01 is localized uniquely in the promoter region conserved in all studied species (Figure 5). On the other hand, the motif I$STAT92E_01 is absent in *D. ananassae* and in species that are not members of the melanogaster group, while *D. melanogaster*, *D. simulans* and *D. sechellia* have a second motif for binding to Stat92E (Figure 8). We also constructed a phylogenetic tree using *Gagr* promoter sequences and found that the obtained tree has a topology that fully reflects the known phylogenetic relationships between the studied *Drosophila* species (Figure 7B).

Alignment of the promoter regions of the *Gagr* orthologs of various *Drosophila* species showed that the kayak binding motif was present in the promoter region of the *Gagr* gene before the separation of Sophophora and *Drosophila* (since its localization is the same in all studied species). In addition, there is a binding motif for Stat92E in the melanogaster subgroup, but it is absent in *D. ananassae*. Finally, the second Stat92E motif is found only in a group of related species: *D. melanogaster*, *D. simulans*, and *D. sechellia*.

Thus, based on an analysis of potential regulators of stress-induced activation of *Gagr* and a search for appropriate motifs in various *Drosophila* species, we conclude that Stat92E and kayak are the main candidates for activating *Gagr* under stress. The presence of the binding motif for regulation of the *Gagr* gene by the JNK cascade is conservative among all of the *Drosophila* species studied, but the regulation of the *Gagr* gene expression by the Jak-STAT pathway was probably acquired by the species of the melanogaster group during evolution. It is noteworthy that the JNK-dependent regulation of stress-induced activation of *Gagr* was indirectly confirmed by an experiment on the induction of an immune response by bacterial lipopolysaccharides in the cultures of S2 cells carrying mutations in regulators of stress MAPK/JNK signaling pathways [18].

## 4. Conclusions

*Drosophila* studies allow us to expand our understanding of the role of domesticated genes of retroelements in the host organism. *Drosophila* has two examples of domestication of the *env* and *gag* genes of LTR-retrotransposons — the *Iris* and *Gagr* genes, respectively [17,58]. *Gagr* homologs are found in all sequenced genomes of the genus *Drosophila*; their structure is maintained by stabilizing selection, which indicates the important function of the *Gagr* gene. Earlier, we have shown that, despite a high conservatism of the gene structure as a whole, its individual sections are under the influence of motif selection, as a result of which the products of the *Gagr* gene and its homologs from the melanogaster subgroup evolved towards the acquisition of a transmembrane domain [17]. At the same time, as shown in the present work, the promoter region of the gene underwent evolution, and as a result, binding sites for the transcription factor STAT appeared. This indicates the formation of a novel gene function in the *Drosophila* genome. The data obtained in this work will contribute to our understanding of the evolutionary potential of retrotransposon gene sequences and their role in stress reactions in *Drosophila*. To clarify and resolve issues relating *Gagr* gene evolution, we plan to analyze the evolution of the expression of the *Gagr* gene in response to stress in different species of *Drosophila*.

## Figures and Tables

**Table 1:** Stress agents and controls used in experiments and stress effects.

Stress Agent	Expected Biological Effect	Treatment Control
Ammonium persulfate, 0.1 M	Oxidative stress	Ammonium sulfate, 0.1 M (as a control of salt stress and of ammonium ions)
Oligomycin A, 1 mM (ethanol solution)	Disruption of mitochondrial proteostasis, inhibition of mitochondrial ATP synthase [25,26]	Ethanol, ~5%
Cadmium chloride, 0.1 M	ER stress, unfolded protein response [27,28,29,30,31]	Magnesium chloride, 0.1 M (as a divalent metal salt)
Zeocin, 10 mM (chelated with copper (II) ions)	Initiation of double-strand DNA breaks [32,33]	Copper (II) chloride, 15 mM

**Table 2:** Primers used in quantitative PCR experiments.

Gene	Forward Primer	Reverse Primer
*Gagr*	AACTTCGATGGCAGTGATCC	GCTCATTTGTCGCGTGAAGA
*vir-1*	CGTAAAGAGTGCCATCAT	CGTGTTCCTGCTCCAAATCT
*Relish*	TCATACACACCGCCAAGAAG	CTGTCTCCTGATGCAGTTCC
*upd3*	AACGGCCAGAACCAGGAATC	GAGAGGGCAAACTGGGACAT
*hid*	GGAAGGAAGCGGATAAGGACA	CCCGATGAACTCGACGCTAC
*sid*	GGAAGTGTTCAAGCGAATTG	AGCAGATACAACGTCTGGTG
*Tspo*	CTAGCAGCCACGCTAAGTC	GTTCGATAGGTCGGAAAGC
*Hsp22*	CTTTCACGCCTTCTTCCAC	GTGAGTTTGTAGCCATCCTTG
*alphaTub84D*	GTGCATGTTGTCCAACACCAC	AGAACTCTCCCTCCTCCATA
*RpL40*	CTGCGTGGTGGTATCATTG	CAGGTTGTTGGTGTGTCC
*EloB*	GCACAAACATACACACTCACG	TTTCCTACTTCGCTTGCACC

**Table 3:** Expression stress markers used in experiments.

Gene	Description
***upd3***	Target of JNK signaling cascade and transcription factor AP-1 [36]; ligand that activates Jak-STAT signaling pathway [37]; one of key cytokines in response to various stress factors.
***vir-1***	Prospective target of Jak-STAT signaling pathway [38,39]. Expression of *vir-1* is activated in response to viral infection [19,38,40] and oxidative stress [41,42].
***Rel***	NFkB transcription factor. Stress-induced activation of Rel expression depends on JNK signaling [43].
***Hsp22***	Chaperone; involved in oxidative stress reactions in mitochondria [44]
***Tspo***	Mitochondrial protein; positive regulator of apoptosis; plays important role in oxidative stress [45]
***Sid***	DNase gene activated by oxidative stress [46]
***Hid***	Target of transcription factor foxo; positive regulator of apoptosis [38]; p53 cascade target [47]

**Table 4:** TFBS enrichment analysis for promoter regions of co-expressed genes. Values presented: adjusted fold enrichment (statistically corrected odds ratios with a 99% confidence interval) for weight matrices of stress-associated transcription factors; significant enrichment is marked in red.

PWM_ID	TF	Cluster 1	Cluster 2	Cluster 3	Cluster 4	Cluster 5	Cluster 6
I$KAY_01	**Kay**	**1.24**	**2.84**	0.70	0.76	**1.10**	0.57
I$KAY_02	**Kay**	0.65	0.60	0.75	**1.01**	0.54	0.51
I$JRA_01	**Jra**	0.59	0.66	0.72	**1.03**	0.51	0.54
I$ATF2_01	**Atf2**	0.67	0.74	0.95	**1.03**	0.56	0.48
I$ATF6_02	**Atf6**	0.73	0.63	0.70	**1.09**	0.52	0.56
I$CREBA_02	**CrebA**	0.81	0.70	0.73	**1.19**	0.53	0.57
I$GATAE_01	**GATAe**	0.64	**1.17**	**1.22**	0.65	0.72	0.48
I$GRN_01	**Grn**	0.63	**1.68**	**1.13**	0.69	0.68	0.40
I$PNR_02	**Pnr**	0.63	**1.01**	0.77	0.68	0.56	0.49
I$PNR_03	**Pnr**	0.63	**1.60**	**1.15**	0.66	0.67	0.42
I$SRP_01	**Srp**	0.63	**1.70**	**1.12**	0.71	0.68	0.40
I$MTTFA_01	**Tfam**	0.60	**1.63**	**1.27**	0.62	0.66	0.44
I$DIF_01	**Dif**	0.57	**1.78**	0.81	0.69	0.59	0.27
I$DIF_02	**Dif**	0.60	**1.52**	0.85	0.66	0.61	0.27
I$DIF_03	**Dif**	0.52	**1.02**	0.93	0.68	**1.17**	0.45
I$REL_01	**Rel**	0.60	**1.38**	0.69	0.67	0.72	0.30
I$STAT_01	**Stat92E**	0.56	**1.16**	0.63	0.73	0.37	0.48
I$STAT92E_01	**Stat92E**	0.61	**1.08**	0.63	0.53	0.53	0.38
I$CNC_01	**Cnc**	0.59	0.61	0.73	0.70	0.60	**1.10**
I$HSF_05	**Hsf**	0.57	0.74	0.67	0.64	0.48	**1.06**

## Supplementary Materials

The following are available online at https://www.mdpi.com/2073-4425/11/4/396/s1, Table S1: Nodes and edges of co-expression networks. Table S2: Weight matrices found in the study of promoters co-expressed with *Gagr* genes. File S1: Multiple alignment: Alignment of the promoter regions of the *Gagr* gene and its orthologs.
